# Croupous Rhinits

**Published:** 1895-06

**Authors:** R. H. A. Boyd

**Affiliations:** San Antonio, Texas


For the Texas Medical Journal.

### CROUPOUS RHINITIS.

BY R. H. A. BOYD, A. M., M. D., SAN ANTONIO, TEXAS.

THE following case is interesting not only on account of its rarity, but also on account of its possible etiology.

Any way, the disease should create enough interest as a possibility of its being diphtheritic. Until quite recently the literature of croupous rhinitis has been very scant, and for a time the only descriptions of the disease that one could find was that found in the journals from time to time. It was there regarded to be of syphilitic origin. How or why this idea prevailed seems almost incredible, unless it was that the treatment suggested had some marked effect on the disease.

I will give the history of the case: H. F. W., aged 31, in good health, spent his honeymoon at Lake Superior in August of 1891. The morning of his return he sent for me, saying he had a fearful cold in his head. There was a semi-purulent discharge running from his nose, which kept him busy wiping it. He was sneezing a great deal; he was aching through his body and head; had a slight fever; temperature 101°, and his nose was completely shut up. I saw he was suffering too much for an ordinary cold. I asked him to come to the office in order that I could examine him with a reflected light.

Inspection showed a pearly white membrane covering the lower and middle turbinated bones and also the septum. It was easily detached and left no bleeding patches. I tried to shrink the swollen membrane with cocaine and found it had no effect on it. The tonsils and half arches and posterior wall of the pharynx had also a few patches. I tried to cleanse the nose with Dobell's solution, but found it of very little use. The same result with per-oxide of hydrogen. Then I tried a weak, warm solution of bi-chloride of mercury, which I found most beneficial. I sprayed him ont several times daily, and at the same time got rid of the false membrane by means of cotton on a probe. Seeing that he was much prostrated, I put him on tincture of iron, and in about eight days he was well.

The question naturally arises, had the newly married relations anything to do with the disease?

We know that erectile tissue covers the turbinated bones, and we know also that venery and the menstral flow will often cause this tissue to become congested. Usually the disease is due to a germ, and a recent investigator in the *American Journal of Medical Sciences* has said it is due to the Klebs-Loeffler bacillus. Whether this be true or not, I am not prepared to say, but I will say that I was quite reckless in exposing myself and instruments to the germs of diphtheria if it were diphtheria. Still I am rather inclined to doubt whether it is diphtheritic, and I will cite the following reasons for my position:

Any one who has used the galvanic cautery much in nasal work knows that he will often get a croupous exudate in his burns, and that, too, without any systemic involvement. The Germans say that the croupous exudate in this condition is due to the insufflation of impure water.

Second. The following winter I had under my care a woman who was a consumptive, much run down, and in the seventh month of pregnancy, came down with a double pneumonia. During her pneumonia an exudate, which was croupous, appeared in her throat which I was able to get out in castes. This did not involve the vocal chords. There were two young children continually in the room. None of the children took diphtheria or diphtheritic croup. In Germany a croupous exudate is seen very often following low, grave diseases as typhus and typhoid fever and pneumonia.

In children I believe it impossible to make the differential diagnosis between nasal diphtheria and croupous rhinitis. The culture test is the only one that can decide this point. Bosworth, in his work on Nose and Throat, lays down certain points of difference. At the present time I do not think they can be received. The subject needs further investigation.

